# Speckle Noise Suppression in SAR Images Using a Three-Step Algorithm

**DOI:** 10.3390/s18113643

**Published:** 2018-10-27

**Authors:** Ze Yu, Wenqi Wang, Chunsheng Li, Wei Liu, Jian Yang

**Affiliations:** 1 School of Electronic and Information Engineering, Beihang University, Beijing 100083, China; yz613@buaa.edu.cn (Z.Y.); wenqi_wang@buaa.edu.cn (W.W.); 2 Department of Electronic & Electrical Engineering, University of Sheffield, Sheffield S1 4ET, UK; w.liu@sheffield.ac.uk; 3 School of Electronic Engineering, Xidian University, Xi’an 710071, China; jianyang@xidian.edu.cn

**Keywords:** synthetic aperture radar (SAR), speckle noise, non-local filtering, probabilistic patch-based (PPB)

## Abstract

Speckle noise can reduce the image quality of synthetic aperture radar (SAR) and complicate image interpretation. This study proposes a novel three-step approach based on the conventional probabilistic patch-based (PPB) algorithm to minimize the impact of bright structures on speckle suppression. The first step improves the calculation accuracy of the weight by pre-processing speckle noise with a linear minimum mean-square error filter and reassessing similarity between pixels. In the second step, an iterative method is developed to avoid interfering with bright structures and acquires a more accurate homogeneous factor by adaptively changing the size of the search window. In the final step, the spreading and blurring of bright structures is corrected using a modified bias-reduction technique. Experimental results demonstrate the proposed algorithm has improved performance for both speckle suppression and preservation of edges and textures, evaluated by indicators including the equivalent number of looks, the edge preservation index, the mean, and standard deviation of ratio images.

## 1. Introduction

Synthetic aperture radar (SAR) is a coherent imaging system [1]. Each pixel in SAR images represents the coherent addition of scatterers from a corresponding resolution cell. These scatterers interfere, either constructively or destructively, depending on the phase of the scatterers. As such, the resulting images exhibit bright and dark pixels and are uneven, even for homogeneous regions. This phenomenon is called speckle noise and it often reduces the quality of images and complicates image interpretation [1,2]. This study proposes a novel speckle removal algorithm to not only suppress speckle noise but also preserve edges and textures.

The simplest speckle removal approach is spatial multi-looking [3], which efficiently suppresses speckle noise at the cost of resolution loss. Three types of non-multi-looking processing methods have been proposed to balance spatial resolution and speckle removal performance.

The first is a local spatial filtering method proposed by Lee [4,5,6,7,8]. Representative algorithms include Kuan [9] and maximum a posteriori (MAP) filtering [10]. Such methods have been implemented in the spatial domain based on Bayesian criteria and a speckle model. Although resolution is well-preserved and speckle noise is suppressed, the edges and textures are not maintained because the speckle model is unsuitable for filtering areas containing strong scattering points.

The second approach involves transform-domain filtering methods, such as linear minimum mean-square error (LMMSE) estimation in the wavelet domain [11]. These methods perform multi-scale decomposition on the image, implement filtering to each decomposition image, and reconstruct the despeckling result by fusing sub-images. Since transform domain methods can distinguish edges from homogeneous areas, these techniques can more accurately preserve edges and textures compared to spatial filtering algorithms. However, these techniques are often worse for de-noising homogeneous areas than the following approach.

The third approach is adaptive filtering, which includes methods based on partial differential equations (PDEs) [12] and non-local approaches [13]. This PDE-based approach gradually suppresses speckle noise during iterative processing and is sensitive to edge preservation. However, repeated iterations tend to diminish texture, particularly in SAR images. The non-local methods exploit similar pixels or blocks in images to implement filtering. It obtains the most comprehensive performance in speckle suppression and preservation of edges and textures. The probabilistic patch-based (PPB) algorithm is a representative of nonlocal methods. It was proposed by Deledalle et al. in 2009 [14]. In 2015, they proposed a unified nonlocal framework where bias-reduction was introduced to reduce the spreading of bright structures [15].

Compared with the conventional PPB, the proposed algorithm achieves a more accurate weighting and homogeneous factor to improve the performance of speckle suppression, with a modified bias-reduction method to further balance speckle suppression with the correction of bright structure spreading.

This paper is structured as follows. The conventional PPB algorithm is introduced and analyzed in Section 2. The three-step algorithm is then proposed to compensate for the limitations of these existing techniques in Section 3. Section 4 presents and analyzes corresponding results by comparing the proposed algorithm with conventional PPB, and Section 5 concludes the paper.

## 2. Conventional PPB Algorithm

**Figure 1:**
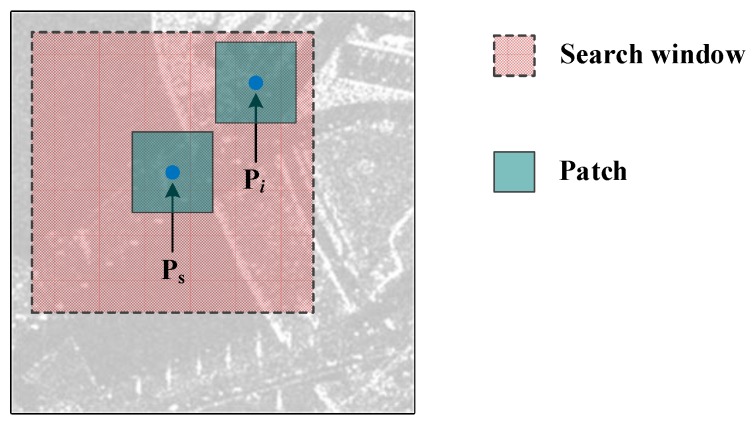
The basic elements in the conventional probabilistic patch-based (PPB) algorithm. P_s_ is the pixel to be processed. The search window and patch are represented by the pink and cyan rectangles, respectively. P*_i_* denotes any pixel in the search window.

As illustrated in Figure 1, P_s_ represents a pixel to be processed in the SAR image. A search window (centered on P_s_) is defined to estimate the intensity of P_s_, as represented by the pink rectangle in the figure. The conventional PPB algorithm calculates the weight $w(P_{s}{,P}_{i})$ between P_s_ and the pixel (P*_i_*) in the search window and replaces the intensity of P_s_ with [14]:

(1) $${\hat{I}}_{P_{s}}=\frac{\sum_{i\in D_{s}}w\left(P_{s},P_{i}\right)I_{P_{i}}}{\sum_{i\in D_{s}}w\left(P_{s},P_{i}\right)}\;$$

where *D_s_* represents a set composed of pixels in the search window and $I_{P_{i}}$ denotes the original intensity of P*_i_*.

This patch region is represented by the cyan rectangle in Figure 1. The weight $w(P_{s},P_{i})$ can be calculated as [14]:

(2) $$w\left(P_{s},P_{i}\right)=\exp\left[-\sum_{k}\frac{2L-1}{h}\log\left(\frac{A_{s,k}}{A_{i,k}}+\frac{A_{i,k}}{A_{s,k}}\right)\right]\;$$

where *A*_s,*k*_ and *A_i_*_,*k*_ are the amplitudes of the *k*th pixels in the two patches centered on P_s_ and P*_i_*, respectively. The greater the weight, the more similar P_s_ and P*_i_*. The term *L* is the equivalent number of looks and *h* is defined as [14]:

(3) $$h=q-E\left[-\sum_{k}\log p\left(A_{s,k},A_{i,k}|I_{s,k}^{*}=I_{i,k}^{*}\right)\right]\;$$

and *q* is given by:

(4) $$q=F_{-\sum_{k}\log p\left(A_{s,k},A_{i,k}|I_{s,k}^{*}=I_{i,k}^{*}\right)}^{-1}\left(\alpha \right)\;$$

where E(⋅) and F(⋅) denote the expectation and cumulative distribution functions, respectively. A bias reduction method was developed to reduce the spreading of bright structures and the intensity of P_s_ was modified as follows [15]:

(5) $${\hat{I}}_{P_{{}_{s}}}^{\mathrm{RB}}={\hat{I}}_{P_{s}}+\alpha_{P_{s}}\left(I_{P_{s}}-{\hat{I}}_{P_{s}}\right)\;$$

where ${\hat{I}}_{P_{s}}^{\mathrm{RB}}$ is the intensity after applying bias reduction and $I_{P_{s}}$ is the intensity of P_s_ in the raw SAR image. The homogeneous factor ($\alpha_{P_{s}}$) corresponding to P_s_ is given by

(6) $$\alpha_{P_{s}}=\max\left(0,1-\frac{{\hat{I}}_{P_{s}}^{2}/L}{\sigma_{P_{s}}}\right)\;$$

and

(7) $$\sigma_{P_{s}}=\frac{\sum_{i\in D_{s}}w\left(P_{s},P_{i}\right)I_{P_{i}}^{2}}{\sum_{i\in D_{s}}w\left(P_{s},P_{i}\right)}-{\hat{I}}_{P_{s}}^{2}\;$$

where $\alpha_{P_{s}}$ is defined on the interval [0, 1]. If P_s_ is in the completely homogeneous area, $\alpha_{P_{s}}$ equals 0. If P_s_ is in the bright structures, $\alpha_{P_{s}}$ tends to 1. A TerraSAR-X image with the resolution of one meter and the processing results acquired by applying the conventional PPB algorithm are shown in Figure 2. Figure 2a displays a raw unquantized single-look image, where the maximum and minimum intensities are 3.68 × 10^7^ and 0, respectively. Figure 2b shows the result processed by Equation (1), and Figure 2c shows the result processed by Equations (1) and (5).

The comparison between Figure 2a and Figure 2b demonstrates the extent of speckle noise suppression achievable with Equation (1). However, the high intensity of the strong scattering targets present in the patches negatively affect the estimation using Equation (1). Figure 2a includes three patches (centered at P_1_, P_2_, and P_3_) with intensities of $I_{P_{1}}=900$, $I_{P_{2}}=601$, and $I_{P_{3}}=345,217$, respectively. The corresponding weights were $w\left(P_{1},P_{2}\right)=0.0255$ and $w\left(P_{1},P_{3}\right)=2.4437\times 10^{-4}$ by applying Equation (2). It is worth noting that $w\left(P_{1},P_{2}\right)>w\left(P_{1},P_{3}\right)$, which indicates that P_2_ is much more similar to P_1_, whereas the product terms satisfy $w\left(P_{1},P_{2}\right)\cdot I_{P_{2}}<w\left(P_{1},P_{3}\right)\cdot I_{P_{3}}$. As a result, the contribution of the dissimilar point (P_3_) is higher when estimating the intensity of P_1_ in Equation (1). This improves the filtering result, which degrades speckle suppression performance. This effect is evident near bright structures, and widens edges and increases the size of strong scattering targets. This effect is referred to as the spreading of bright structures and can be seen in Figure 2b. The performance of speckle suppression can be further improved by considering the impact of bright structures, which will be discussed in Section 3.1.

Equation (5) was used to correct for the spreading of bright structures by moderately restoring the original intensities of pixels according to the factor $\alpha_{P_{s}}$, as shown in Figure 2c. However, speckle noise was also restored, particularly near bright structures. This occurred because of the inverse relationship between speckle suppression and the spreading correction in Equation (5). The value of $\alpha_{P_{s}}$ obtained from Equation (6) w typically close to 1 for pixels near bright structures. As such, ${\hat{I}}_{P_{s}}^{\mathrm{RB}}$ tends to $I_{P_{s}}$ in Equation (5), which indicates the processed results are similar to the original image and the speckle remains mostly unaffected. We investigated this limitation using two approaches.

**Figure 3:**
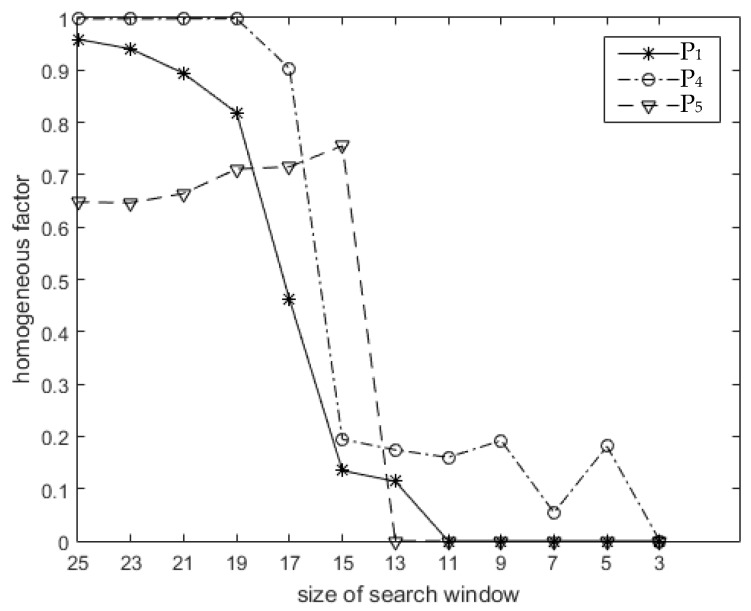
A variation in the homogeneous factor with a size matching the search window. The three curves correspond to P_1_, P_4_, and P_5_ in Figure 2a. The initial window size was 25 and the step size for the window reduction was 2.

**Figure 4:**
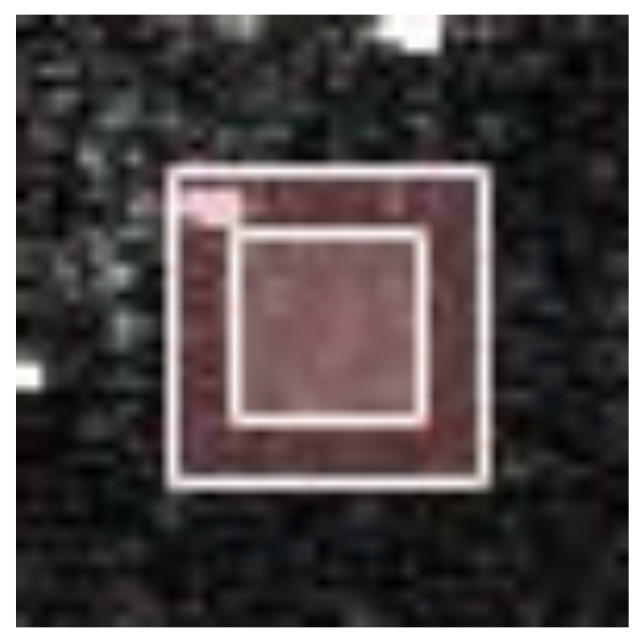
Two search windows centered on P_4_ with outer and inner frame sizes of 25 and 15, respectively. A bright structure is evident between these two frames, which did not affect the homogeneous factor calculated using Equation (6) in the inner frame. Homogenous factors of 0.9032 and 0.1942 were produced by the large and small windows, respectively.

The first approach involved calculation of a homogeneous factor $\alpha_{P_{s}}$. There are three search windows centered on P_1_, P_4_, and P_5_ in Figure 2a. These three points were located in homogeneous areas, and we set the size of the search window to 25 results in $\alpha_{P_{1}}=0.9591$, $\alpha_{P_{4}}=0.9977$, and $\alpha_{P_{5}}=0.6476$. As the size of the search window decreased, a sudden decrease occurred in the homogeneous factor, as shown in Figure 3. For example, as the size of the search window centered on P_4_ decreased from 17 to 15, the homogeneous factor decreased from 0.9032 to 0.1942. This occurred because bright structures were excluded from the search window, as shown in Figure 4. Therefore, a more accurate value of $\alpha_{P_{s}}$ could be determined by choosing an appropriately-sized search window to avoid interfering with bright structures. This process is discussed further in Section 3.2. The second approach involves modifying the form of Equation (5) to balance speckle suppression with the correction of bright structure spreading, which will be discussed in Section 3.3.

## 3. Three-Step Algorithm for Speckle Suppression

**Figure 5:**
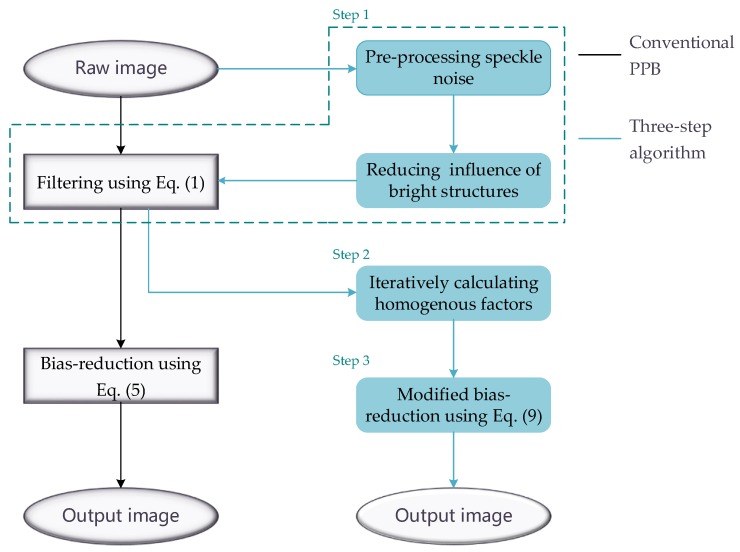
An illustration of the three-step algorithm.

Figure 5 compares the proposed three-step algorithm with the conventional PPB algorithm. The conventional PPB algorithm applies Equations (1) and (5) to the raw image. In the proposed algorithm, the first step improves the calculation accuracy of the weight by pre-processing speckle noise and reducing the effects of bright structures, and better effect of speckle suppression can be obtained using Equation (1). In the second step, an iterative method is utilized to obtain a more accurate value of $\alpha_{P_{s}}$ by adaptively changing the size of the search window. The final step corrects for spreading and blurring of bright targets using a modified bias-reduction method.

### 3.1. Speckle Pre-Processing and Weight Correction

The primary objective of speckle pre-processing is to suppress speckle noise in homogeneous areas without losing edge and texture details, which reduces the influence of speckle noise on weight calculation. This study adopts the linear minimum mean-square error (LMMSE) filter for pre-processing [11]. Although the denoising results produced by this algorithm are not ideal, it is highly suitable for preserving edges and textures.

Then, a threshold was set, which was 25 dB higher than the average intensity of the search window [16]. Any pixels with an intensity exceeding this threshold were considered to be strong scattering points. The influence of these points on weight calculation was then considered in four cases, as demonstrated in Figure 1.

Case 1: Patches centered on P_s_ and P*_i_* do not contain any strong scattering points, which indicates that an influence of strong scattering points on weight calculation does not exist. In this case, the weight $w(P_{s}{,P}_{i})$ was calculated using Equation (2).

Case 2: Both P_s_ and P*_i_* are strong scattering points. It was assumed that these two points are likely similar. The weight was then calculated using Equation (2).

Case 3: Either P_s_ or P*_i_* was a strong scattering point (not both). In this instance, the two patches centered on P_s_ and P*_i_* were thought to be completely different and the weight was accordingly set to 0.

Case 4: The patches centered on P_s_ or P*_i_* contained strong scattering points, none of which were P_s_ or P*_i_*. In order to reduce the impact of the strong scattering points, the weight was then determined from Equation (2), in which all intensities for strong scattering points were replaced by the average intensity of the patch.

### 3.2. Iteratively Calculating the Homogeneous Factor

As illustrated in Figure 3, the homogeneous factor $\alpha_{P_{s}}$ is dependent on the size of the search window. Therefore, the simplest approach to improving the accuracy of $\alpha_{P_{s}}$ was to reduce the window size. However, this also reduces the number of similar pixels and degrades speckle suppression performance in homogeneous areas. As such, an iterative method was developed to adaptively maximize the search window without affecting the accuracy of $\alpha_{P_{s}}$. The details of this process are as follows.

(1) The initial side length of the search window centered on P_s_ is set to $\Delta_{S_{0}}$ and the corresponding homogeneous factor $\alpha_{S_{0}}$ is calculated using Equation (6). Bright structures have little effect on this calculation. If $\alpha_{S_{0}}$, the estimation of the homogeneous factor for P_s_ is less than 0.5, which is an empirical threshold. In this case, $\alpha_{S_{0}}$ is the final estimation. Otherwise, the iteration continues. This step can reduce the computational complexity by identifying pixels that require homogeneous factor correction.

(2) Let $\Delta_{S_{i}}=\Delta_{S_{i-1}}-2$ (*i* = 1, 2, …), and the corresponding homogeneous factor $\alpha_{S_{i}}$ is determined using Equation (6). If $\Delta_{S_{i}}\times \Delta_{S_{i}}$ is less than the minimal size of the search window (i.e., 3 × 3), the iteration terminates and $\alpha_{S_{i}}$ represents the final estimation. Otherwise, the process continues to step (3).

(3) The ratio $r_{1}$ is calculated as:

$$r_{1}=\alpha_{S_{i}}/\alpha_{S_{i-1}}\;$$

The value of $\alpha_{S_{i}}$ decreases dramatically if the region does not contain any bright structures, as illustrated in Figure 3. Therefore, if $r_{1}$ is less than 0.5, indicating the homogeneous factor is less than half the previous value, the iteration terminates and $\alpha_{S_{i}}$ is the final estimation. Otherwise, the process continues to step (2) when *i* equals 1, or step (4) when *i* is greater than 1.

(4) The ratio $r_{2}$ is calculated as:

$$r_{2}=\alpha_{S_{i}}/\alpha_{S_{i-2}}\;$$

The iteration terminates if $r_{2}$ is less than 0.5 and $\alpha_{S_{i}}$ becomes the final estimation, as in the previous step. Otherwise, the process returns to step (2).

### 3.3. Correcting the Spreading and Blurring of Bright Targets

As aforementioned, the minimum size of the search window was set to 3 × 3. Therefore, homogeneous factors were updated by applying the methods proposed in Section 3.2., with the exception of 3 × 3 regions surrounding bright structures. A modified bias-reduction method is proposed to reduce the spreading of these bright structures.

A new ratio $r_{3}$ can be defined as:

(8) $$r_{3}=\frac{{\hat{I}}_{P_{s}}}{I_{P_{s}}}\;$$

which indicates whether significant spreading occurs or not. Equation (5) can be modified to balance speckle suppression with the correction of bright structure spreading as follows:

(9) $${\hat{I}}_{P_{s}}^{\mathrm{RB}}={\hat{I}}_{P_{s}}+F(\alpha_{P_{s}},\;r_{3})(I_{P_{s}}-{\hat{I}}_{P_{s}}),\;\alpha_{P_{s}}\in [0,1],F(\alpha_{P_{s}},r_{3})\in [0,1]\;$$

where $F(\alpha_{P_{s}},r_{3})$ satisfies the following conditions:

(1) When $r_{3}\leq 1$,

(10) $$F(\alpha_{P_{s}},r_{3})=0\;$$

Equations (9) and (10) demonstrate that ${\hat{I}}_{P_{s}}^{\mathrm{RB}}$ equals ${\hat{I}}_{P_{s}}$ in areas that do not exhibit bright structure spreading. The level of speckle suppression is maintained in such areas.

**Figure 6:**
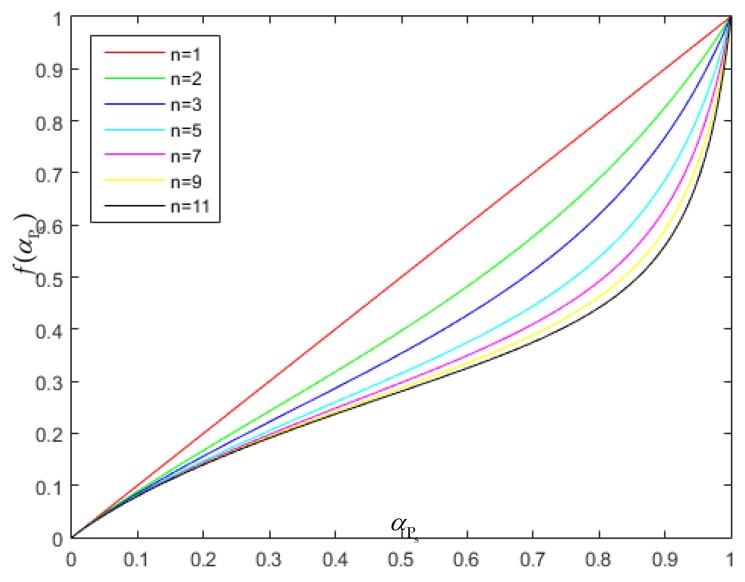
Variations in $f(\alpha_{P_{s}})$ with $\alpha_{P_{s}}$ and *n*. $f(\alpha_{P_{s}})$ is defined in Equation (12). $\alpha_{P_{s}}$ is the homogeneous factor and *n* is a parameter to balance speckle suppression with the correction of bright structure spreading.

(2) When $r_{3}>1$, indicating the presence of spreading, the following condition is satisfied:

(11) $$F(\alpha_{P_{s}},r_{3})=(1-\frac{1}{r_{3}})\alpha_{P_{s}}+\frac{1}{r_{3}}f(\alpha_{P_{s}})\;$$

where

(12) $$f(\alpha_{P_{s}})=\alpha_{P_{s}}^{\frac{n}{n-(n-1)\alpha_{P_{s}}}}\;$$

when $0<\alpha_{P_{s}}<1$, $f(\alpha_{P_{s}})$ is less than $\alpha_{P_{s}}$, as illustrated in Figure 6, where *n* is a parameter to balance speckle suppression with the correction of bright structure spreading. In the conventional PPB algorithm, $F(\alpha_{P_{s}},r_{3})=\alpha_{P_{s}}$, which corrects for the spreading of bright structures but degrades speckle noise suppression, as discussed in Section 2. In contrast, for $F(\alpha_{P_{s}},r_{3})=f(\alpha_{P_{s}})$, ${\hat{I}}_{P_{s}}^{\mathrm{RB}}$ tends to ${\hat{I}}_{P_{s}}$, which improves the performance of speckle suppression but induces obvious bright structure spreading. Equation (11) makes $f(\alpha_{P_{s}})\leq F(\alpha_{P_{s}},r_{3})\leq \alpha_{P_{s}}$, which results in more balanced performance with some suppression of both spreading and speckle.

**Figure 7:**
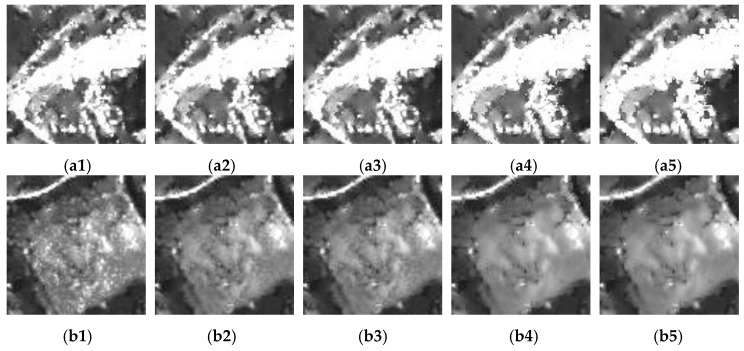
Impact of *n* in Equation (12) on the speckle suppression and correction for the spreading of bright structures. There are two groups of experimental results: (**a1**) to (**a5**) and (**b1**) to (**b5**). For (**a1**) and (**b1**), *n* is set to 1. And for the second, third, fourth, and fifth columns, the values of *n* are 5, 10, 20 and 50, respectively.

Figure 7 demonstrates the impact of *n* on the speckle suppression and correction for the spreading of bright structures. From left to right, the values of *n* for these images are 1, 5, 10, 20, and 50. When *n* = 1, the speckle noise in the corresponding image was the most serious. As *n* increased, the speckle noise was more effectively suppressed, while the spreading of bright structures worsened. When the value of *n* was between 5 and 10, a more balanced performance was obtained. In this study, the value of *n* was set to 5.

In the filtering process described by Equation (9), the bright structures are also suppressed and blurred. A matrix denoted by $\alpha_{\mathrm{final}}$ was developed to recover these structures. This matrix is the same size as the image, and each element in this matrix corresponds to the homogeneous factor of a pixel. Bright structures in SAR images can be positioned from the matrix $\alpha_{\mathrm{final}}$ by the canny operator, after which ${\hat{I}}_{P_{s}}^{\mathrm{RB}}$ is directly set to the original intensity of these bright structures.

## 4. Experimental Results and Analysis

**Figure 8:**
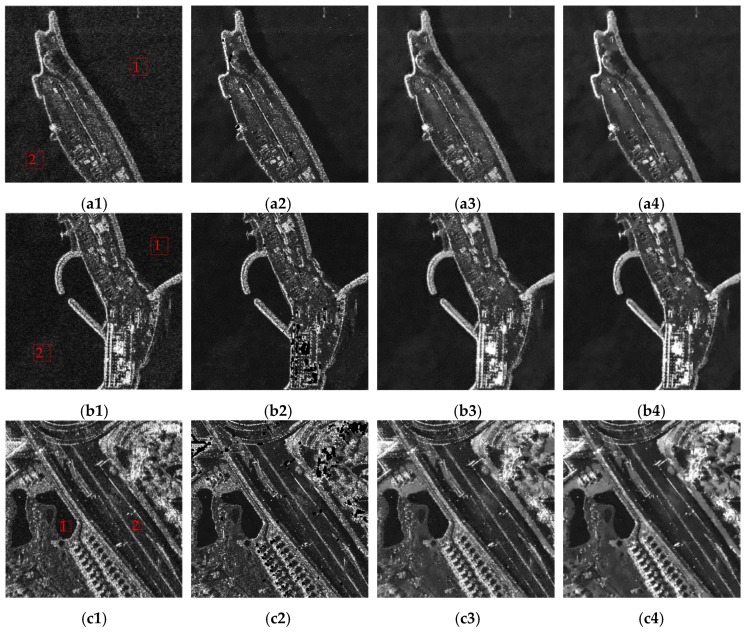

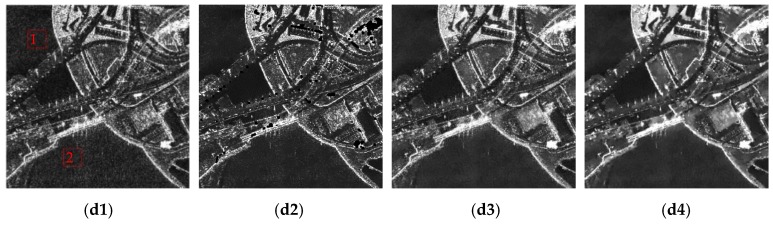
Despeckling results. (**a1**), (**b1**), (**c1**) and (**d1**) show raw SAR images. (**a2**), (**b2**), (**c2**) and (**d2**) illustrate results obtained by the fast non-local algorithm. (**a3**), (**b3**), (**c3**) and (**d3**) illustrate results obtained by the conventional PPB algorithm. (**a4**), (**b4**), (**c4**) and (**d4**) illustrate results obtained by the proposed three-step algorithm.

Four TerraSAR-X images were used to validate the proposed algorithm, as illustrated in the first column of Figure 8. Among these, Figure 8a1 and Figure 8b1 exhibit clear edges and uniform backgrounds, whereas Figure 8c1 and Figure 8d1 include complex structures. All these images contain strong scattering points. These characteristics help demonstrate the comprehensive performance of the proposed technique. The results achieved using the fast non-local means algorithm [17], conventional PPB algorithm, and proposed three-step algorithm are shown in the second, third, and fourth columns of Figure 8, respectively.

Several quantitative metrics were used to evaluate Figure 8: the equivalent number of looks (ENL) [18], the edge preservation index (EPI) [19], the mean $\mu_{r}$, and the standard deviation $\sigma_{r}$ of the ratio image [20,21]. The results of this evaluation are presented in Table 1. The terms ENL_1_ and ENL_2_ were calculated using the areas enclosed by the red frames, labeled 1 and 2, respectively.

Processing and evaluation results indicated that all three algorithms significantly suppress speckle noise. The fast non-local and conventional PPB algorithms have basically the same ability in speckle suppression, which is indicated by the ENL value. The fast non-local algorithm performed the worst in edge preservation. The proposed algorithm produced the highest ENL and EPI values, indicating that it was most successful in both preserving edges and suppressing speckle.

**Figure 9:**
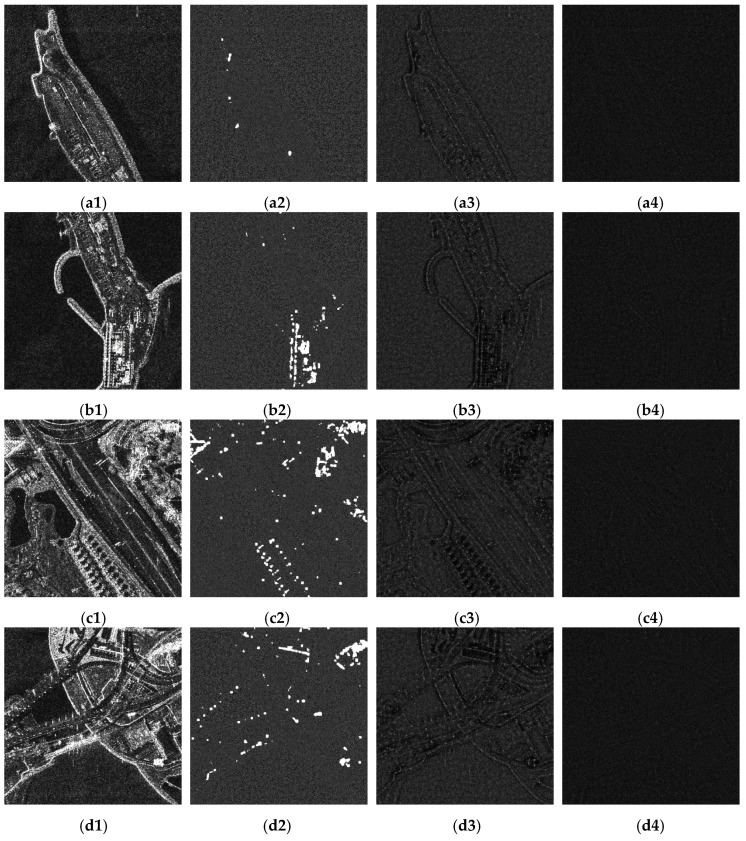
Ratio images. (**a1**), (**b1**), (**c1**) and (**d1**) show raw SAR images. (**a2**), (**b2**), (**c2**) and (**d2**) show ratio images corresponding to the fast non-local means algorithm. (**a3**), (**b3**), (**c3**) and (**d3**) illustrate ratio images corresponding to the conventional PPB algorithm. (**a4**), (**b4**), (**c4**) and (**d4**) represent ratio images corresponding to the proposed algorithm.

A point-to-point comparison of the texture preservation results is shown in Figure 9. These images were produced using the ratio between raw and de-speckled data, with corresponding evaluation results shown in the last two columns of Table 1. The application of an ideal despeckling algorithm would produce a ratio image containing only speckle points, indicating that the mean and standard deviation of the ratio image would be 1 and $\sqrt{1/L}$, respectively, for an *L*-look raw image [3]. As all raw SAR images in this study were single-look complex images, the ideal mean and standard deviation were both one. As shown in the second column of Figure 9, the ratio images obtained by the fast non-local algorithm contained bright structures, so the mean and standard deviation of the ratio images were far from one. Ratio images corresponding to the conventional PPB algorithm are shown in the third column of Figure 9. They contain obvious geometric structures related to the original images, indicating that not only speckle noise but also textures were removed by the conventional PPB algorithm. In contrast, the ratio images produced using the proposed technique exhibited much weaker geometric structure, as shown in the fourth column. This indicates that the proposed algorithm can preserve texture details, with a mean and standard deviation of ratio images closer to one compared with the conventional PPB algorithm. These results demonstrate the superior performance of the proposed method.

## 5. Conclusions

In this study, we developed a novel three-step technique based on the conventional PPB algorithm. The proposed algorithm improved the calculation accuracy of the weighting by pre-processing speckle noise with the LMMSE filter and reducing the influence of bright structures. The algorithm also improves upon the accuracy of the homogeneous factor by adaptively changing the size of the search window, and then corrects for the spreading and blurring of bright structures. TerraSAR-X images with clear edges, uniform backgrounds, and complicated internal structures were used to validate this technique. This algorithm has the advantages of the conventional PPB and has better performance for both speckle suppression and the preservation of edges and textures. In a future study, deep neural networks, such as generative adversarial networks, which have adaptive and strong filtering abilities, will be used to further improve the performances. In particular, we expect that suppressing bright structure spreading can be achieved without weakening the denoising effect.

## Figures and Tables

**Figure 2:**
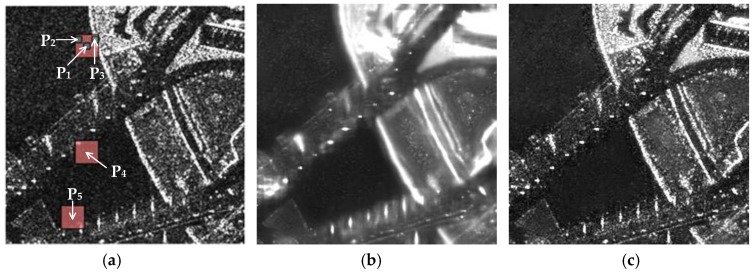
Processing results achieved using the conventional PPB algorithm: (**a**) the raw single look complex image, (**b**) the result processed using Equation (1), and (**c**) the result processed by Equations (1) and (5). (**a**) There are three search windows centered on P_1_, P_4_, and P_5_, and three patches centered at P_1_, P_2_, and P_3_.

**Table 1:** Evaluation results.

Algorithm	Image	ENL_1_	ENL_2_	EPI	$\mu_{r}$	$\sigma_{r}$
Raw image	1	0.9996	0.9682	--	--	--
Fast non-local algorithm		12.7594	12.3647	0.5134	1.5448 × 10^10^	1.4475 × 10^12^
Conventional PPB		16.9518	12.7141	0.8685	0.8648	0.6390
Three-step algorithm		36.3338	26.3064	0.9484	0.9484	0.8271
Raw image	2	0.9983	1.0154	--	--	--
Fast non-local algorithm		22.1223	4.8051	0.2603	7.9030×10^10^	1.9913×10^12^
Conventional PPB		17.3786	15.0505	0.8278	0.8473	0.6132
Three-step algorithm		40.2398	26.0787	0.9435	0.9458	0.7977
Raw image	3	1.042	1.0051	--	--	--
Fast non-local algorithm		17.5744	2.5683	0.2936	3.3912 × 10^11^	1.4966 × 10^13^
Conventional PPB		10.7677	4.651	0.9180	0.8055	0.4402
Three-step algorithm		67.2727	36.5582	0.9480	0.9631	0.8314
Raw image	4	1.0044	1.0147	--	--	--
Fast non-local algorithm		15.7269	11.3674	0.3532	3.1675 × 10^11^	9.9027 × 10^12^
Conventional PPB		13.2521	12.7311	0.9174	0.8292	0.4846
Three-step algorithm		33.0672	47.3125	0.9491	0.9598	0.8227

ENL_1_ and ENL_2_ represent the equivalent number of looks calculated using the areas enclosed by the red frames, labeled 1 and 2, in Figure 8. EPI represents the edge preservation index. $\mu_{r}$ and $\sigma_{r}$ are the mean and standard deviation of ratio images shown in Figure 9.
